# Rates and Predictors of Malignancy in Bethesda III and IV Thyroid Nodules: A Prospective Study

**DOI:** 10.7759/cureus.76615

**Published:** 2024-12-30

**Authors:** Eman Z Azzam, Marwa A Salah, Waleed A Aboelwafa, Rawan M Essam, Maha E Bondok

**Affiliations:** 1 Internal Medicine, University of Alexandria, Alexandria, EGY; 2 Head and Neck Surgery, Alexandria University Teaching Hospital, Alexandria, EGY; 3 Internal Medicine, Alexandria Main University Hospital, Alexandria, EGY

**Keywords:** bethesda category iii, bethesda category iv, bethesda risk cytopathological classification, fnac, indeterminate nodules, post-thyroidectomy histopathology, thyroid nodules, ti-rads

## Abstract

Aim: Thyroid nodules, based on high-resolution ultrasonography (HRUS), are among the most common endocrine abnormalities that affect the general population because of their high estimated prevalence rates. Fine needle aspiration cytology (FNAC) is a safe, cost-effective modality to differentiate between benign and malignant thyroid nodules based on the Bethesda System for Reporting Thyroid Cytopathology (BSRTC), thus avoiding unnecessary surgery. However, categories III and IV of BSRTC remain a controversial issue in clinical practice, encompassing a wide range of risks of malignancy. Hence, our study aimed to assess the malignancy rates of thyroid nodules classified as Bethesda III and IV categories as evidenced by post-thyroidectomy histopathology; study the association between the American College of Radiologists Thyroid Image Reporting and Data System (ACR-TIRADS) score of these two categories and the postoperative histopathological analysis; and study the predictors of malignancy in these two categories.

Materials and methods: A prospective study was conducted on 242 patients who underwent FNAC throughout the study from December 2022 to August 2023. All patients who performed FNAC were primarily subjected to history taking, clinical examination, thyroid-stimulating hormone (TSH), thyroid autoantibodies (antithyroglobulin (anti-TG) and thyroid peroxidase antibodies (TPO Abs)), and HRUS with a further categorization of thyroid nodules according to the ACR-TIRADS scoring system. The cytological aspirates were categorized according to the BSRTC. Patients with Bethesda III and IV categories were resorted to surgery according to clinical factors, sonographic features, and patients’ preferences.

Results: A total of 17 cases with Bethesda III and 65 patients with Bethesda IV were included. Seventy-one out of 82 patients (86.6%) underwent surgical intervention. The proportions of malignant nodules classified as TIRADS-2, TIRADS-3, TIRADS-4, and TIRADS-5 scores were 0.0, 4.5 (n=1/22), 22.7 (n=5/22), and 72.7% (n=16/22), respectively. The rate of malignancy was 18.2% (n=2/11) among class III and 33.3% (n=20/60) among class IV-categorized Bethesda thyroid nodules. In univariate logistic regression analysis, age ≥ 40 years, body mass index ≥ 30 kg/m², higher TSH, positive anti-TG antibodies, radiation exposure, irregular borders, marked hypoechogenicity, ill-defined margins, microcalcifications, solid consistency, taller than wide growth, solitary nodule, and nodule size > 2 cm, and suspicious lymph nodes were associated with higher malignancy risk. In multivariate regression analysis, positive anti-TG Abs, radiation exposure, irregular borders, taller-than-wide growth, hypoechogenicity, calcifications, and solid consistency remain to be independent predictors of malignancy.

Conclusion: The malignancy rates of Bethesda class III and IV nodules in this study met the estimated malignancy risk proposed by BSRTC. TIRADS scores 4 and 5 confer a higher risk of malignancy in Bethesda III and IV thyroid nodules. Positive thyroglobulin antibodies and radiation exposure are independent factors of malignancy in Bethesda III and IV nodules. Moreover, ultrasound features, including irregular borders, taller-than-wider growth, hypoechogenicity, calcifications, and solid consistency, are associated with increased malignancy risk and should be considered in the surgical selection of patients.

## Introduction

Thyroid nodules are quite a common entity in the field of endocrinology with a high prevalence rate. Whereas detectable nodules based on clinical palpation range from 5% to 7% of the adult population, studies have shown that 20-67% of the general population harbors thyroid nodules based on high-resolution ultrasonography (HRUS). Although most thyroid nodules are benign, asymptomatic, and clinically insignificant, the clinical importance of thyroid nodules lies in the ability to exclude thyroid cancer. It has been estimated that the prevalence of thyroid cancer is 5-15% of detected thyroid nodules [1]. The higher prevalence of thyroid cancer as compared to previously reported figures is mostly attributed to overdiagnosis with the advance of technology on the one hand and to the increased exposure to potential risk factors and the emergence of hidden risk factors on the other hand.

Different endocrine associations have set standardized systems for reporting thyroid ultrasound based on the constellation of radiological features to identify sets of nodule characteristics associated with risk levels for malignancy. Moreover, these classification systems aim to guide practitioners to the necessity of fine-needle aspiration cytology (FNAC) based on the malignant potentials of thyroid nodules with translated risk of malignancy [2-4].

Bethesda System for Reporting Thyroid Cytopathology (BSRTC) provides better communication among clinicians, where it offers standardized nomenclature for the interpretation of thyroid fine needle aspirates. BSRTC describes six diagnostic categories of lesions: non-diagnostic/unsatisfactory, benign, atypia of unknown significance (AUS), suspicious for follicular neoplasm (SFN), suspicious for malignancy (SM), and malignant. The six diagnostic categories of the Bethesda system have individual implied risks of malignancy that influence management paradigms and avoid unnecessary intervention [5].

Although FNAC is a rapid, safe, and cost-effective diagnostic tool in the approach of thyroid nodules, it has its own limitations [3]. The first limitation is that approximately 20% of FNAC yield inconclusive results categorized as being indeterminate, as they don’t provide clear guidance as regards the management. Indeterminate cytology includes Bethesda categories III, IV, and V, with a total malignancy risk ranging between 6-30%, 10-40%, and 45-75%, respectively [6]. This wide variation of the risk of malignancy has led to unnecessary surgeries to avoid a possible case of cancer or an underestimation of the probability of missing a true case of cancer. These limitations paved the way for novel diagnostic markers to refine the estimated malignancy risk based on cytopathology. Hence, the development of thyroid molecular markers, although it is still unavailable in most of the centers [7]. The second limitation is that despite FNAC having high diagnostic accuracy in diagnosing thyroid nodules with a reported sensitivity and specificity of 65-98% and 72-100%, respectively, false-positive and/or false-negative results can occur for multiple causes, the most important of which is related to the quality of the aspirated sample [8]. Moreover, the FNAC may lack the ability to accurately diagnose special circumstances such as nodules less than 1 cm, thyroiditis, cases of follicular neoplasm (FN), and cases where malignancy couldn't be accurately justified [9].

Our study aimed to assess the malignancy rates of thyroid nodules classified as Bethesda III and IV indicated by histopathology after thyroidectomy and to study the predictors of malignancy in the implicated thyroid nodules. Moreover, we aimed to study the association between the American College of Radiologists Thyroid Image Reporting and Data System (ACR-TIRADS) of the studied nodules and final histopathology results.

## Materials and methods

A prospective study was conducted at the endocrine outpatient clinic at the internal medicine department and endocrine unit at the Alexandria Main University Hospital (AMUH), a tertiary referral center, in Alexandria, Egypt. Patients presented with thyroid nodules were recruited during the study period, from the beginning of December 2022 till the end of August 2023. All enrolled individuals underwent detailed thyroid examination, thyroid-stimulating hormone (TSH), anti-thyroglobulin antibodies (anti-TG Abs), and thyroid peroxidase antibodies (TPO Abs) testing. HRUS was performed by the same sonographer with a full description of thyroid nodules, including number, size, echogenicity, composition, borders, margins, presence of calcifications, vascularity, and suspicious lymph nodes. The ACR-TIRADS scoring system further characterized thyroid nodules. ACR-TIRADS is a standardized scoring system as proposed by the American College of Radiologists, in which thyroid nodules are categorized into five categories, TR1 to TR5, assigned as benign, not suspicious, mildly suspicious, moderately suspicious, and highly suspicious, respectively, based on the sum of points given for each thyroid nodule according to its features, namely composition, echogenicity, shape, margins, and the presence or absence of echogenic foci. ACR-TIRADS recommends fine needle aspiration cytology (FNAC) for TR3-5 thyroid nodules according to their size threshold [10]. Cases presented with pure cystic nodules or those with nodules less than 1 cm with no suspicious malignancy features (absence of extrathyroidal extension or suspicious lymphadenopathy) were excluded from the study. Ultrasound-guided FNAC was performed according to ACR-TIRADS guidelines. In cases with multiple thyroid nodules, FNAC was taken from the nodules with the most suspicious sonographic features. BSRTC further assessed cytological aspirates into six categories. Patients aspirated who met categories I and III based on BSRTC were submitted to repeat FNAC unless they refused or lost follow-up. Patients classified as Bethesda III and IV were resorted to surgical intervention based on patients’ risk factors, including age, sex, family history of thyroid cancer, history of previous head and neck irradiation, sonographic features, and patient preference. A correlation between ultrasound features based on the ACR-TIRADS classification system of cases with Bethesda III and IV and the final histopathology of the surgical specimens was made. Predictors of malignancy for patients with categories III and IV were assessed (predictors include age, sex, family history of thyroid cancer, history of ionizing radiation, BMI, TSH level, TPO Abs, anti-TG Abs, and ultrasound parameters of thyroid nodules, including size, shape, margins, composition, echogenicity, calcification, vascularity, and the presence of suspicious lymph nodes).

Written informed consent was taken from all patients enrolled in this study for their approval, with a full explanation of all procedures done, including both FNAC and thyroidectomy, together with a detailed explanation of the possible hazards of these interventions.

Statistical analysis

Data was analyzed using IBM SPSS Statistics for Windows, Version 27 (Released 2020; IBM Corp., Armonk, New York, United States). Qualitative data were described using numbers and percentages, while quantitative data were described using range (minimum-maximum), mean, standard deviation (SD), median, and interquartile range (IQR). The chi-square test, Fisher’s exact test, and likelihood ratio were performed to compare the categorical variables among groups. The normal distribution of numerical data (quantitative variables) was explored using the Shapiro-Wilk test. Accordingly, the student t-test was used to compare the means of normally distributed variables, while the Mann-Whitney U-test was applied to compare the mean ranks of abnormally distributed variables. The significance of the obtained results was judged at the 5% level with p ≤ 0.05.

## Results

A total number of 242 patients underwent FNAC during the study period. The studied population included 194 (80.2%) female and 48 male patients (19.8%). The mean age was 45.4 ± 11.1 years. Eighteen patients (7.4%) had a positive family history of thyroid cancer, and 34 patients (14%) reported previous radiation exposure. The mean TSH was 2.6 ± 1.8 mIU/L; of a total of 242, euthyroid patients represented most of the cases (149, 61.6%). Almost 38.4% (93) had hypothyroidism.

**Figure 1:**
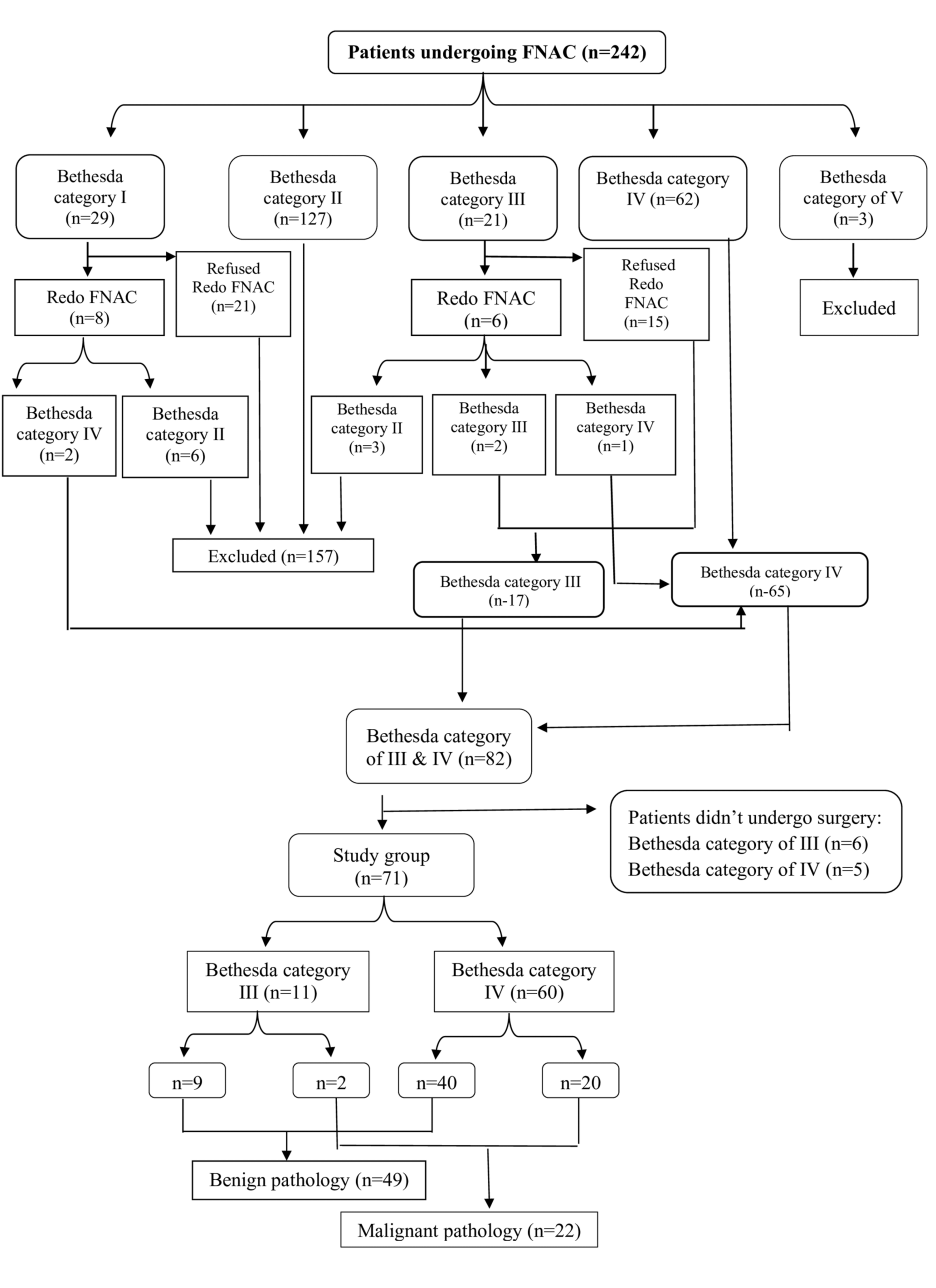
Flowchart of the total number of patients undergoing FNAC over the study period FNAC: fine needle aspiration cytology; n=number

The resulting histopathology was classified according to the Bethesda classification system in 2017. The frequency of each Bethesda class was as follows: Bethesda I was 12% (29); Bethesda II was 52.5% (127); Bethesda III was 8.7% (21); Bethesda IV was 25.6% (62); Bethesda V was 1.2% (3). FNAC was repeated for eight patients with Bethesda class I (ND) nodules, yielding two samples with category IV and six with class II (benign). Another six patients with class III underwent repeat FNAC; two remained as such, one turned class IV, and three turned benign (class II) (Figure 1).

**Figure 2:**
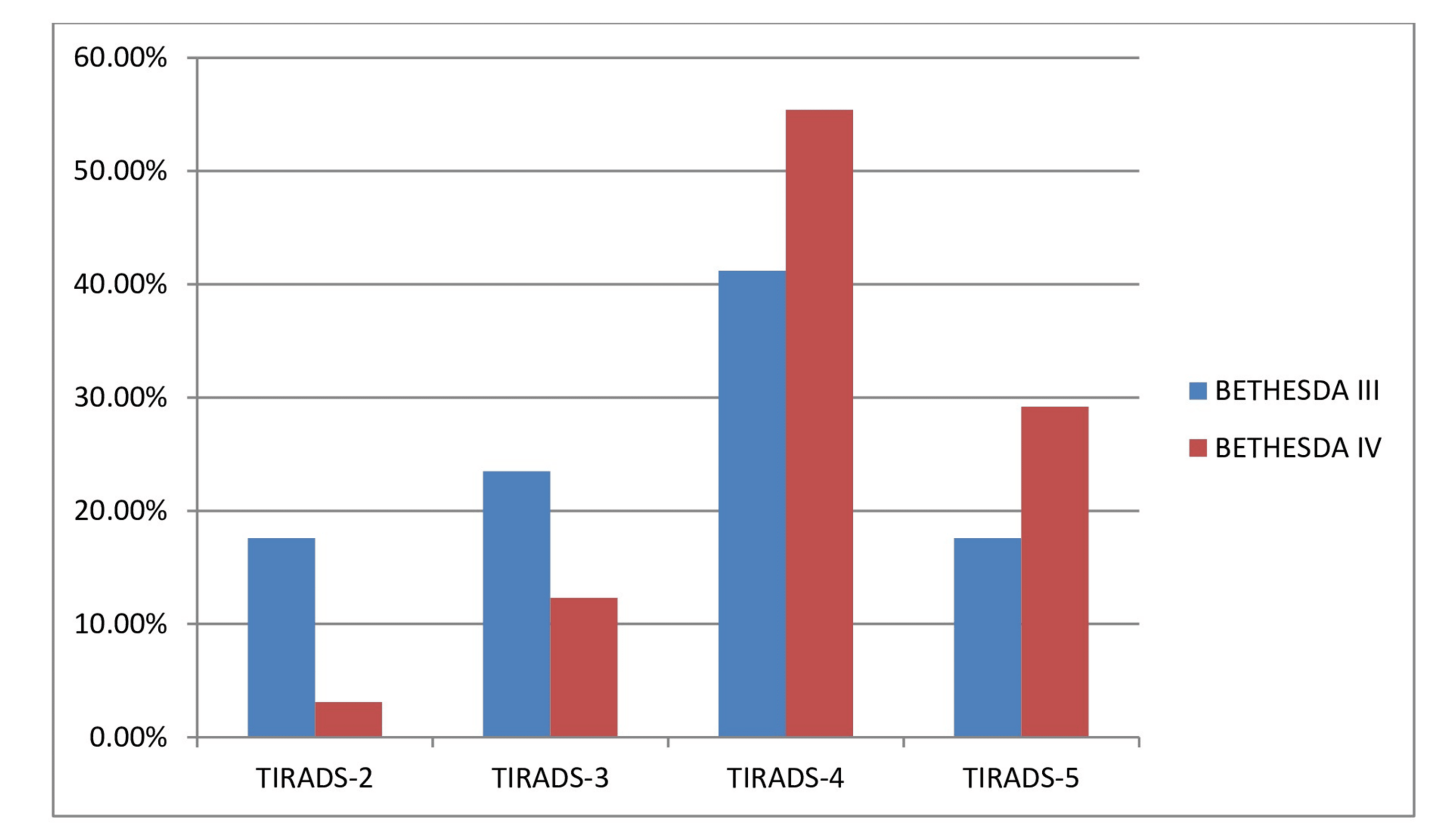
TIRADS score of Bethesda III and IV categorized thyroid nodules TIRADS: Thyroid Imaging Reporting and Data System; No: number; %: percentage

Eighty-two patients were categorized as Bethesda III and IV. Based on patients’ risk factors and preferences, 71 patients (71 of 82) underwent thyroidectomy; 60 cases with Bethesda IV and 11 cases with Bethesda III (Figure 1). Among patients with thyroid nodules classified as Bethesda III, 3 of 17 cases (17.6%) were categorized as TIRADS-2 by HRUS, 4 of 17 (23.5%) were categorized as TIRADS-3, 7 of 17 (41.2%) were classified as TIRADS-4, and 3 of 17 (17.6%) nodules were given a TIRADS-5 score. In cases with thyroid nodules classified as Bethesda IV, most of the nodules fell under TIRADS-4 (36 of 65, 55.4%) and TIRADS-5 (19 of 65, 29.2%). Only 2 of 65 (3.1%) cases were categorized as TIRADS-2, and 8 of 65 (12.3%) cases were assigned as TIRADS-3 (Figure 2).

The overall malignancy rate among Bethesda III and IV patients was 31%, as determined by post-operative histopathological analysis. The malignancy rate was 18.2% for Bethesda III and 33.3% for Bethesda IV. The most common malignant lesion was papillary thyroid carcinoma, identified in 18 of 22 cases (81.82%) (one-third of these were papillary microcarcinoma), followed by follicular carcinoma (3 of 22, 13.64%) and a single case of medullary thyroid cancer (1 of 22, 4.55%) (Table 1).

**Table 1:** Malignancy rates of Bethesda III and IV categorized thyroid nodules B: Bethesda; X^2^: Pearson's chi-square test; n: number

Histopathology	Patients with B III and IV thyroid nodules who underwent surgery (n=71)				Total	
	B IV (n=60)		B III (n=11)			
	No	Percentage	No	Percentage	No	Percentage
Malignant	20	33.3	2	18.2	22	31
Benign	40	66.7	9	81.8	49	69
c^2^=0.998 (p=0.318)						

Association between thyroid histopathology and various clinical and ultrasonographic characteristics of Bethesda categories III and IV

Among 71 patients with Bethesda classes III and IV, the female sex was more represented among malignant nodules (90.9%) than benign ones (71.4%), yet this difference was not statistically significant. The mean age of patients with malignant nodules was significantly higher (53.9 ± 12.4 years) compared to those with benign nodules (44.3 ± 13.5 years) (p=0.006).

The median size of the malignant nodules was 2.1 cm (IQR: 1.8-3.2 cm). The ultrasonographic features of the malignant nodules were as follows: 13 of 22 nodules (59.1%) were solid, 11 of 22 nodules (50%) had irregular borders, and 17 of 22 nodules were hypoechoic; among these, 4 of 22 nodules (18.2%) were markedly hypoechoic and 13 of 22 (59.1%) were moderately hypoechoic. Eight of 22 nodules (36.4%) exhibited microcalcifications, 6 of 22 nodules (27.3%) had rim or eggshell calcifications, and 10 of 22 patients (45.5%) had suspicious lymph nodes.

Concerning patients with Bethesda III categorized thyroid nodules who underwent surgery, three cases showed nuclear atypia, five cases showed architectural atypia, two cases had both nuclear and architectural atypia, and only one case showed Hurthle cell atypia (n=11). Postoperative histopathology revealed papillary thyroid cancer in the two patients with Bethesda III categorized thyroid nodules; one case showed nuclear atypia, and the other case showed both nuclear and architectural atypia.

Demographic data, thyroid status, thyroid autoantibodies, and ultrasound features of both benign and malignant nodules based on postoperative histopathology in patients with Bethesda III and IV categorized thyroid nodules are presented in Table 2.

**Table 2:** Demographic and sonographic features of Bethesda III and IV thyroid nodules in relation to postoperative final histopathology n: number, p-value: test of significance; OR: odds ratio; UL: upper limit; LL: lower limit; CI: confidence interval; BMI: body mass index; TSH: thyroid-stimulating hormone; Anti-TG Abs: antithyroglobulin antibodies; TPO Abs: thyroperoxidase antibodies; STN: solitary thyroid nodule; cm: centimeter; >: more than; ≥: more than or equal to The significance of the obtained results was judged at the 5% level with p≤0.05.

Variable	Postoperative pathology		p-value
	Benign (n=49)	Malignant (n=22)	
Age			
≥40 years	27	19	
<40 years	22	3	0.011
Sex			
Female	35	20	
Male	14	2	0.122
BMI			
≥30 kg/m^2^	20	15	
<30 kg/m^2^	29	7	0.033
Family history of thyroid cancer			
Yes	0	4	
No	49	18	0.008
Exposure to radiation			
Yes	4	7	
No	45	15	0.028
Consistency			
Hard	0	12	
Soft	49	10	0.001
Palpable lymph nodes			
Yes	10	13	
No	39	9	<0.001
Thyroid status			
Euthyroid	37	18	
Hypothyroid	12	4	0.551
TSH (mIU/ml)			
Mean	1.4±1.1	2.3±1.2	0.033
Anti TG Abs			
Positive	9	16	
Negative	40	6	<0.001
Anti-TPO Abs			
Positive	15	12	
Negative	34	10	0.29
Echogenicity			
Markedly hypoechoic	2	4	
Hypoechoic	19	13	
Isoechoic	21	5	
Hyperechoic	7	0	0.009
Calcifications			
Microcalcifications	11	8	
Rim/eggshell of calcifications	3	6	
Absent	35	8	0.01
Consistency			
Solid	15	13	
Cystic/mixed	34	9	0.023
Margins			
Ill-defined	13	12	
Well-defined	36	10	0.008
Borders			
Irregular	10	11	
Regular	39	11	0.012
Number of nodules			
Solitary	12	13	
Multiple	37	9	0.005
Shape			
Tall more than wide	9	12	
Wide more than tall	40	10	0.043
Suspicious lymph nodes			
Yes	11	12	
No	38	10	0.05
Vascularity			
Absent	19	4	
Central/mixed	12	9	
Peripheral	18	9	0.179
Nodule size			
<2 cm	38	11	
≥2 cm	11	11	0.02

Correlation between TI-RADS and postoperative histopathology of Bethesda III and IV classified thyroid nodules

In the current study, four patients bearing thyroid nodules were classified as TIRADS 2 by HRUS, two cases were Bethesda III and two were Bethesda IV by FNAC. None of them turned back to be malignant as evidenced by the post-operative pathology. On the other hand, eight nodules were classified as TIRADS 3, two of them were Bethesda III and six were Bethesda IV by FNAC. Only one case categorized as Bethesda IV was shown to be malignant in the postoperative pathology specimen. Forty nodules were classified as TI-RADS 4, five of them were Bethesda III, 35 were Bethesda IV by FNAC, and five were assigned by postoperative histopathology as being malignant; one case was Bethesda III and four cases were Bethesda IV. Finally, 19 nodules were classified as TI-RADS 5, with most of them being malignant according to postoperative histopathology (16 of 19, 84%). One case was Bethesda III, and 15 cases were Bethesda IV (Table 3).

**Table 3:** Correlation between TIRADS and postoperative histopathology of Bethesda III and IV classified thyroid nodules B III: Bethesda class III; B IV: Bethesda class IV; TIRADS: Thyroid Imaging Reporting and Data Reporting System; %: percentage

TIRADS score	Number	Malignant (B III)	Benign (B III)	Malignant (B IV)	Benign (B IV)
2	4 (5.6%)	0 (0%)	2 (22.2%)	0 (0%)	2 (5%)
3	8 (11.3%)	0 (0%)	2 (22.2%)	1 (5%)	5 (12.5%)
4	40 (46.3%)	1 (50%)	4 (44.4%)	4 (20%)	31 (77.5%)
5	19 (26.8%)	1 (50%)	1 (11.1%)	15 (75%)	2 (5%)
Total	71 (100%)	2 (100%)	9 (100%)	20 (100%)	40 (100%)

The proportion of malignant nodules classified as TIRADS-2, TIRADS-3, TIRADS-4, and TIRADS-5 scores were 0.0%, 4.5%, 22.7%, and 72.7%, respectively.

Regarding the role of the TIRADS scoring system as a diagnostic test for patients with Bethesda III and IV. TIRADS showed 66.7% sensitivity and 93.9% specificity in total cases of Bethesda III and IV. The sensitivity and specificity of the TIRADS scoring system were 75% sensitivity and 95% specificity for Bethesda IV cases, and 50% sensitivity and 88.9% specificity for Bethesda III cases (Table 4).

**Table 4:** Performance of TIRADS classification as diagnostic test for malignancy in Bethesda III and IV thyroid nodules B: Bethesda; TIRADS: Thyroid Imaging Reporting and Data System; PPV: positive predictive value; NPV: negative predictive value; %: percentage

TIRADS classification	Sensitivity (%)	Speciﬁcity (%)	PPV	NPV	Accuracy
B III and IV	66.7	93.9	84.2	88.5	87.3
B IV	75	95	88.2	88.4	88.3
B III	50	88.9	50	88.9	81.8

Predictors of malignancy

Univariate logistic regression analysis showed that the predictors for malignancy among Bethesda III and IV nodules were age ≥ 40 years (OR=5.2, 95% CI 1.3-19.7, p=0.011), BMI ≥30 kg/m^2^ (OR=3.1, 95% CI 1.1-9.0, p=0.033), family history of thyroid cancer (OR=3.7, 95% CI 2.5-3.5, p=0.008, history of radiation exposure (OR=5.3, 95% CI 1.3-20.5, p=0.028), hard consistency (OR=5.9, 95% CI 3.4-10.4, p=0.001), palpable lymph nodes (OR=5.6, 95% CI 1.9- 16.9, p<0.001), higher TSH (p=0.033), positive antithyroglobulin (anti-TG) antibodies (OR=11.9, 95% CI 3.6-38.7, p≤0.001), marked hypoechogenicity p=0.009), microcalcifications (p=0.005), solid consistency (OR=3.3, 95% CI 1.2-9.3, p=0.023), ill-defined margins (OR=4, 95% CI 1.4-11.6, p=0.008), irregular borders (OR=3.9, 95% CI 1.3-11.6, p=0.012), solitary thyroid nodule (OR=4.5, 95% CI 1.5-13, p=0.005), taller than wide growth (OR=3.1, 95% CI 1.1-9.4, p=0.043), suspicious lymph nodes (OR=2.9, 95% CI 1.0-8.4, p=0.05), and nodule size >2 cm (OR=3.5, 95% CI 1.2-10.1, p=0.02), were associated with higher malignancy risk. Multivariate regression analysis showed that positive anti-TG antibodies (OR=5.5, p=0.002), radiation exposure (OR=4.7, p=0.013), irregular borders (OR=4.1, p=0.031), taller than wide growth (OR=4, p=0.007), hypoechogenicity (OR=3.7, p=0.019), calcifications (OR=3, p=0.024), and solid consistency (OR=2.7, p=0.043) remain to be associated with increased risk of malignancy among Bethesda III and IV categorized thyroid nodules (Tables 5-6).

**Table 5:** Univariate regression analysis of significant predictors of malignancy of Bethesda III and IV thyroid nodules n: number; p-value: test of significance; OR: odds ratio; UL: upper limit; LL: lower limit; CI: confidence interval; BMI: body mass index; TSH: thyroid-stimulating hormone; anti-TG Abs: antithyroglobulin antibodies; TPO Abs: thyroperoxidase antibodies; STN: solitary thyroid nodule; cm: centimeter; >: more than; ≥: more than or equal to The significance of the obtained results was judged at the 5% level with p≤0.05.

Predictor	OR (LL-UL 95% CI)	p-value
Age (≥40 years)	5.2 (1.3-19.7)	0.011
Sex		0.122
BMI (≥30 kg/m^2^)	3.1 (1.1-9.0)	0.033
Diabetes		0.077
Family history of thyroid cancer	3.7 (2.5-3.5)	0.008
Exposure to radiation	5.3 (1.3-20.5)	0.028
Hard consistency	5.9 (3.4- 10.4)	0.001
Palpable lymph nodes	5.6 (1.9-16.9)	<0.001
Thyroidal status		0.597
TSH (mIU/L)		0.033
Positive anti-TG Abs	11.9 (3.6-38.7)	<0.001
Positive TPO Abs		0.290
Marked hypo echogenicity		0.009
Microcalcifications		0.005
Solid composition	3.3 (1.2-9.3)	0.023
Ill-defined margin	4.0 (1.4-11.6)	0.008
Irregular border	3.9 (1.3-11.6)	0.012
STN	4.5 (1.5-13.0)	0.005
Taller than wide	3.1 (1.1-9.4)	0.043
Vascularity		0.179
Suspicious lymph nodes	2.9 (1.0-8.4)	0.050
Nodule size > 2 cm	3.5 (1.2-10.1)	0.020

**Table 6:** Multivariate regression analysis of significant predictors of malignancy of Bethesda III and IV thyroid nodules p-value: test of significance; anti-TG: antithyroglobulin; CI: confidence Interval The significance of the obtained results was judged at the 5% level with p≤0.05.

Predictor	Adjusted odds ratio (95% CI) (AOR)	p-value
Positive anti-TG antibodies	5.5 (2.1-18.3)	0.002
Exposure to radiation	4.7 (2.4-15.7)	0.013
Irregular border	4.1 (1.3-13.5)	0.031
Taller than wide	4.0 (1.8-14.2)	0.007
Hypo-echogenicity	3.7 (1.1-11.8)	0.019
Calcifications	3.0 (1.6-12.1)	0.024
Solid consistency	2.7 (1.2-9.6)	0.043
Model sensitivity=91.5%		

## Discussion

Thyroid nodules are highly prevalent endocrine disorders owing to the widespread diagnostic modalities. Despite being commonly met in clinical practice, it is seldom of clinical significance. The main challenge lies in the ability to exclude thyroid cancer, which is reported in 5-15% of patients depending on multiple risk factors and the population studied [1]. Thus, relying on accurate diagnostic tools to better characterize malignancy risk and avoiding unnecessary biopsies and surgeries remains prudent. HRUS provides precise delineation of thyroid nodules [3]. Commonly reported ultrasound features of malignancy include microcalcification, irregular margins, taller growth, hypoechogenicity, intra-nodular vascularity, and suspicious lymph nodes, each bearing different sensitivities and specificities to identify thyroid cancer [2,3]. Accordingly, different endocrine associations have proposed an ultrasound-based malignancy risk stratification for thyroid nodules, guiding practitioners to perform FNAC based on a constellation of sonographic features and translated risk of malignancy [2-4]. The BSRTC is the most popular classification for reporting thyroid FNAC. Categories III, IV, and V of the BSRTC are considered grey areas of intermediate malignancy risk. Hence, the recent BSRTC system has advocated using molecular markers to identify genetic mutations/rearrangements, especially in patients classified as categories III and IV, representing approximately one-quarter of thyroid nodule aspirates. This would potentially identify patients who are truly candidates for surgery [11].

It has been observed that the rates of malignancy among the most controversial Bethesda classes, namely class III and IV, vary among different institutions, mostly due to variability in the randomization of patients, interpretation of thyroid specimens by pathologists, surgical referral, and geographical distribution. The reported malignancy rates for Bethesda class III and IV in different studies range from 10% to 30% for AUS/follicular lesions of unknown significance (FLUS) and 25-40% for FN/SFN [12]. Yet these rates are difficult to predict if not treated by surgical intervention. Here, we present the association between FNAC and histopathological results in patients with categories III and IV who were resorted to thyroidectomy. We demonstrated that the total malignancy rate within Bethesda classes III and IV was 31%. The rates of malignancy in our results were 18.2% in Bethesda III and 33.3% in Bethesda IV, comparable to the rates originally estimated by the Bethesda system. In our work, the malignancy rates are higher for Bethesda class IV (33.3%) than for Bethesda III (18.2%) without statistically significant differences.

In agreement with our results, Yaprak Bayrak and Eruyar found that the malignancy rates among 108 patients with Bethesda III and 47 patients with Bethesda IV who underwent surgery were 25% and 27.6%, respectively, with no significant differences between categories (p=0.67) [12]. Similarly, in line with the reported rates in the literature, Godoi Cavalheiro et al. reported a malignancy rate of 16% among thyroid nodules classified as Bethesda category III and 17% among those classified as Bethesda category IV [13].

However, from a clinical point of view, the increased percentage of malignancy in categories III and IV of the Bethesda scale in some studies cannot be ignored and should be taken into consideration while dealing with these categories. Higher rates of malignancy were described by Chirayath et al., who studied 176 consecutive nodules, where they reported malignancy rates of 54.6% and 72.4% for Bethesda classes III and IV, respectively [14]. Moreover, Zahid et al. reported malignancy in Bethesda category III and Bethesda category IV thyroid nodules in 29.6% and 47.1% of cases, respectively [15].

Thyroid nodules classified as Bethesda III exhibit a relatively low risk of malignancy. The usual way to manage this diagnostic modality is to repeat the FNAC, which may reclassify the nodules in most cases, or to resort to molecular testing to support ruling in or out of malignancy. In centers where molecular testing isn’t feasible, physicians may choose between active surveillance versus diagnostic surgery based on clinical criteria and sonographic features [16]. However, studies specifically designed to assess the risk of malignancy show higher than reported malignancy rates. Ho et al. reported a malignancy risk ranging between 26.6% and 37.8% among patients with Bethesda III thyroid nodules [17]. Moreover, a wide range of rates of malignancy was observed by Horne et al. in 106 nodules with Bethesda class III, further subclassified into microfollicular architecture (corresponding to FLUS) and nuclear atypia (corresponding to AUS), with reported malignancy rates of 7% and 56%, respectively [18]. Moreover, Mathur et al. recommended surgical intervention in patients with thyroid nodules classified as Bethesda III subclassified as AUS with the presence of focal nuclear atypia, focal microfollicular proliferation, and focal Hurtle cell proliferation as shown to be associated with higher rates of malignancy rates [19]. Similarly, Mosca et al. demonstrated higher malignancy rates for Bethesda class IIIB (AUS) as compared to Bethesda class IIIA (FLUS) [20]. Accordingly, provided the higher malignancy rates in Bethesda III thyroid nodules than that originally predicted by the Bethesda System, surgical resection is recommended in certain cytologic conditions.

Another important consideration that should be kept into account while dealing with cases classified as Bethesda III nodules yielding the same results on repeated FNAC. In our study, six cases with Bethesda III underwent repeated FNAC, where half of these patients turned out to be benign, one case turned out to be Bethesda IV, and two cases remained classified as Bethesda III nodules. In a study by Rosario, on 150 Bethesda III-categorized thyroid nodules undergoing repeated FNAC, almost half of the cases remained as such (48.6), 36% turned out to be benign, almost 7% of cases were recategorized as Bethesda IV and 7% turned out to be SM; Bethesda V with only 1.3% yielded non-diagnostic results. The rate of malignancy after repetition was 22.6%, and they observed that different malignancy rates were found when applying the use of combined ultrasonography and FNAC, where the highest malignancy rates were found in ultrasound-suspicious AUS subcategory Bethesda III nodules and the least in non-ultrasound-suspicious FLUS subcategory of Bethesda III nodules [21]. Lourdes et al. found in their study that 64.4% of Bethesda III nodules who underwent repeated FNAC had a malignancy outcome with non-significant differences as regards clinical, ultrasound, and cytopathologic features of malignant and benign nodules in the final postoperative histopathology, suggesting that surgery may be a reasonable option in such cases [22].

Diagnostic lobectomy is usually the most adopted protocol for managing cases with Bethesda IV; however, molecular testing can be offered to refine malignancy risk before resorting to surgery after taking into consideration the clinical profile of the patients together with the sonographic features. Studies dedicated to studying the risk of malignancy in Bethesda IV are sparse. A retrospective observational Italian study was performed to evaluate the risk of malignancy in patients with Bethesda IV nodules who underwent surgery. The estimated risk of malignancy in their work was 28.8%, and the working team recommended lobectomy rather than total thyroidectomy in category IV for nodules ≥4 cm as found to be associated with higher malignancy rate and a higher propensity of lymphatic and/or vascular invasion and higher probability of subsequent therapeutic radioactive ablation [23].

Thus, considering the high risk of malignancy in patients harboring thyroid nodules classified as Bethesda III and IV observed in different studies, and as molecular markers are not yet widely available. Assessing the patient's clinical characteristics and sonographic features of thyroid nodules is of utmost importance in decision-making to avoid the underestimated risk of malignancy on one side or unnecessary surgery on the other side.

Thyroid Imaging Reporting and Data System (TIRADS) is a standardized scoring system first proposed by Horvath et al. in 2009. It depends on various ultrasound features allowing practitioners to stratify thyroid nodules into five categories based on the risk of malignancy, with TIRADS-1 and 2 showing the lowest risk and TIRADS-5 carrying the highest risk of malignancy. Moreover, TIRADS categorization recommends either follow-up or proceeding to FNAC based on the given score [24].

In our work, considering all Bethesda III and IV nodules that underwent thyroidectomy, the proportions of malignant nodules classified as TIRADS-2, TIRADS-3, TIRADS-4, and TIRADS-5 were 0.0%, 4.5%, 22.7%, and 72.7%, respectively. By testing the accuracy of the TIRADS-5 scoring system for prediction of malignancy in total cases categorized as Bethesda III and IV nodules, the TIRADS score had a sensitivity of 66.7%, specificity of 93.9%, positive predictive value of 84.2%, and NPV of 88.5%. For Bethesda III cases, the TIRADS score had a sensitivity of 50 and a specificity of 75%. For Bethesda IV, the TIRADS score had a specificity and sensitivity of 88.9% and 95%, respectively. The PPV and NPV of TIRADS score for categories III and IV by BSTRC were 50%, 88.9%, 88.2%, and 88.4%, respectively. The low sensitivity and PPV of TIRADS score in the prediction of malignancy in cases of Bethesda III is mostly related to the small sample size for cases with Bethesda III. Most of our malignant cases in the Bethesda IV category met sonographic features of TIRADS-5 score (15/20) of 75%, with a malignancy rate of 88.2%. None of the cases with a TIRADS score of 2 in this category were malignant. Five percent (1/20) of post-thyroidectomy-proven malignant cases had a TIRADS score of 3, and (4/20) 20% had a TIRADS score of 4. It has been shown that the malignancy rates among TIRADS 3 and 4 cases of the Bethesda IV category are almost comparable, mostly attributed to the small sample size.

Periakaruppan et al. studied ultrasound features of 184 nodules among an Indian population using the TIRADS scoring system and correlated the results of TIRADS and the Bethesda classification system. The working team found that most of the nodules, 117/184, had a TIRADS score of 2, none of which were malignant, and the risk of malignancy among nodules with TIRADS 3, 4, and 5 was 2.2%, 38.5%, and 77.8%, respectively. Moreover, they observed a good correlation between TIRADS and Bethesda classification of the studied nodules [25]. Huang et al. studied the concordance between TIRADS and the Bethesda reporting system and final histopathology post-surgery in 446 patients. He observed a high reproducibility of TIRADS score 3 in ruling out cancer with good concordance with Bethesda classification and histopathology. While TIRADS scores 4 and 5 nodules exhibited low positive predictive value with the Bethesda reporting system. However, the TIRADS score had a good PPV with post-surgery histopathology [26].

By investigating the predictors of malignancy in Bethesda III and IV-classified thyroid nodules, we found that age equal to or more than 40 years, higher body mass index (BMI), history of previous radiation exposure, higher TSH, positive anti-TG antibodies, and sonographic features including the presence of solitary nodules, nodules with size >2 cm, marked hypoechogenicity, irregular borders, microcalcifications, ill-defined margins, and the presence of suspicious lymph nodes were significantly associated with increased malignancy risk. However, after adjustment of variables, positive thyroglobulin antibodies, history of radiation exposure, and among ultrasonographic features; irregular borders, taller than wide growth, hypo-echogenicity, calcifications, and solid consistency remain significant predictors of malignancy.

In agreement with our work, age ≥40 years was reported by Ryu et al. as a predictor factor of malignancy in Bethesda III-classified thyroid nodules and by Baloch et al. in Bethesda IV-classified thyroid nodules [27,28]. 

Another retrospective study included 127 cases with Bethesda IV thyroid nodules. The rate of malignancy was 38.6%. They concluded that using a TSH level with a cutoff value ≥2.1 mU/l and age ≥47 years has a sensitivity of 44.9% and a specificity of 76.9% in the prediction of malignancy in Bethesda IV nodules [29]. 

We illustrated that anti-TG antibodies are associated with a 5.7-fold increase in the risk of thyroid cancer in Bethesda III and IV nodules. Thus, it could be an added parameter in decision-making surgical intervention in these categories. Similarly, a meta-analysis included 17 studies on thyroglobulin antibodies, involving a total number of 34,488 patients. It found that there is an elevated risk of thyroid cancer in anti-TG positive versus anti-TG negative patients [30].

Concerning the suspicious ultrasound features most likely to predict malignancy in Bethesda categories III and IV in our study, we found that the presence of irregular borders (p=0.031), taller-than-wide growth (p=0.007), hypoechogenicity (p=0.019), calcifications (p=0.024), and solid consistency (p=0.43) were significantly associated with the risk of malignancy. Compatible with our findings, Li et al. concluded that hypoechogenicity, microcalcification, irregular borders, and a taller-than-wide shape increased the risk of thyroid cancer in Bethesda III and IV nodules, with hypoechogenicity associated with an almost two-fold increased risk of thyroid cancer and microcalcification is coupled with triple risk [31]. In another Saudi study designed to assess the malignancy rates and predictors of malignancy among 187 Bethesda III thyroid nodules who underwent surgical excision, Alshahrani and his team found by analysis of US features that malignancy was significantly associated with nodules with irregular margins, microcalcification, multiple numbers, and hypoechogenicity; however, no statistical difference was found among benign and malignant nodules regarding nodule size and internal vascularity [32]. On the other hand, Hacim et al. found in their observational study involving 159 patients with indeterminate nodules who had further undergone total thyroidectomy that hypoechogenicity, solitary nodules, and solid structures, but not calcifications, were associated with malignancy in Bethesda III, IV, and V thyroid nodules [33]. Whereas Alyusuf et al. found that hypoechogenicity and calcifications are independent predictors of malignancy in patients diagnosed with Bethesda III and IV thyroid nodules on FNAC). In contrast, the size of the dominant nodules, number of nodules, irregular borders, taller-than-wide shape, and the presence of lymph nodes were comparable between benign and malignant nodules [34].

In summary, the malignancy rate was 18% in Bethesda III and 33% in Bethesda IV thyroid nodules, meeting the estimated malignancy risk proposed by BSRTC. Considering all Bethesda III and IV nodules, the proportions of malignant nodules classified as TIRADS-2, TIRADS-3, TIRADS-4, and TIRADS-5 scores were 0.0%, 4.5%, 22.7%, and 72.7%, respectively. Positive anti-TG antibodies are associated with a 5.5-fold, and previous exposure to radiation was associated with a 4.7-fold increased risk of malignancy. Moreover, hypoechogenicity, calcifications, irregular borders, taller-than-wide growth, and solid consistency are coupled with significantly higher malignancy risk in Bethesda III and IV; thus these factors should be kept in consideration in the selection of candidates for surgical intervention.

Thus, according to the results of the present study, we recommend proper assessment of patients with Bethesda III and IV thyroid nodules with TIRADS-4 and 5 scores with solid composition, hypoechoic echotexture, taller than wide growth, irregular margins, and the presence of calcification, particularly among patients with a history of previous irradiation and/or the presence of positive anti-TG antibodies. These parameters should be kept in consideration to avoid unnecessary surgeries.

Recommendations

We recommend extending the study period to yield more cases and to design more studies in our region to identify the most significant predictors of malignancy, especially sonographic features, to justify surgical intervention for patients with thyroid nodules classified as Bethesda III and IV categories.

Strengths and limitations of the study

The main strength of this study is the prospective design of the study. Moreover, the same ultrasonographer performed the total cases recruited in this work. navigating any interobserver variability Additionally, we categorized patients with thyroid nodules according to the TIRADS scoring system, a commonly adopted system used in clinical practice. The main limitation of the study is the sample size due to the time allocated for this study.

## Conclusions

The malignancy rates of Bethesda class III and IV nodules in this study met the estimated malignancy risk proposed by BSRTC. TIRADS scores 4 and 5 confer a higher risk of malignancy in Bethesda III and IV thyroid nodules. Positive thyroid antibodies and radiation exposure are independent factors of malignancy in Bethesda III and IV nodules. Moreover, sonographic features including irregular borders, taller-than-wider growth, hypoechogenicity, calcifications, and solid consistency are associated with increased malignancy risk and should be taken into account in the surgical selection of patients.
